# Integration of Sentinel-1 and Sentinel-2 for Classification and LULC Mapping in the Urban Area of Belém, Eastern Brazilian Amazon

**DOI:** 10.3390/s19051140

**Published:** 2019-03-06

**Authors:** Paulo Amador Tavares, Norma Ely Santos Beltrão, Ulisses Silva Guimarães, Ana Cláudia Teodoro

**Affiliations:** 1 Postgraduate Program in Environmental Sciences, State University of Pará (UEPA), 66095-100 Belém, Brazil; paulo.tavares@uepa.br (P.A.T.); normaely@uepa.br (N.E.S.B.); 2 Operations and Management Center of the Amazon Protection System (CENSIPAM), 66617-420 Belém, Brazil; ulisses.silva@sipam.gov.br (U.S.G.); 3 Earth Sciences Institute (ICT) and Faculty of Sciences (FCUP), University of Porto, 4169-007 Porto, Portugal; amteodor@fc.up.pt (A.C.T.)

**Keywords:** machine learning, random forest, spatial analysis, optical data, radar data, urban land cover

## Abstract

In tropical regions, such as in the Amazon, the use of optical sensors is limited by high cloud coverage throughout the year. As an alternative, Synthetic Aperture Radar (SAR) products could be used, alone or in combination with optical images, to monitor tropical areas. In this sense, we aimed to select the best Land Use and Land Cover (LULC) classification approach for tropical regions using Sentinel family products. We choose the city of Belém, Brazil, as the study area. Images of close dates from Sentinel-1 (S-1) and Sentinel-2 (S-2) were selected, preprocessed, segmented, and integrated to develop a machine learning LULC classification through a Random Forest (RF) classifier. We also combined textural image analysis (S-1) and vegetation indexes (S-2). A total of six LULC classifications were made. Results showed that the best overall accuracy (OA) was found for the integration of S-1 and S-2 (91.07%) data, followed by S-2 only (89.53%), and S-2 with radiometric indexes (89.45%). The worse result was for S-1 data only (56.01). For our analysis the integration of optical products in the stacking increased de OA in all classifications. However, we suggest the development of more investigations with S-1 products due to its importance for tropical regions.

## 1. Introduction

Land Use and Land Cover (LULC) data are important inputs for countries to monitor how their soil and land use are being modified over time [1,2]. It is also possible to identify the impacts of increasing urban environments in different ecosystems [3,4,5,6], monitoring protected areas, and the expansion of deforested areas in tropical forests [1,7,8,9].

Remote sensing data and techniques are used as tools for monitoring changes in environmental protection projects reducing in most cases the prices of surveillance. An example is the LULC approach for monitoring Reduced Emissions from Deforestation and Forest Degradation (REDD+) [10,11] and for ecosystem services (ES) modeling and valuation [12,13,14]. For the latter purpose, the LULC mapping has been used to enhance the results found for Costanza et al. [15] that provided global ES values. In that research, the values have been rectified since its first publication [16,17]; the LULC approach provides land classes which allow to estimate ES by unit area, making it possible to extrapolate ES estimates and values for greater areas and biomes around the world by using the benefit transfer method [1,17,18]. Nonetheless, Song [19] highlights the limitation of these values estimations, since LULC satellite-based products have uncertainties related to the data.

From an Earth Observation (EO) perspective, it is desirable to have free and open data access, e.g., Landsat and Sentinel families [20]. These are orbital sensors which, when combined (Landsat-5–8 and the optical sensor of the Sentinel family, the Sentinel-2, hereafter S-2), will provide a 3-day revisit time on the same point on the Earth surface [21,22]. In contrast, these sensors have a medium-quality spatial resolution (30 m for the Landsat and 10 m for the S-2) when compared with other data, such as the Quickbird (0.6 m) and Worldview (0.5 m), but can deliver satisfactory results when the correct methodology is applied [23,24,25].

More recently, the United States (US) started to consider reintroduce prices for the Landsat products acquisition [26]. That is a step backwards when we consider that more than 100,000 published articles were produced since 2008 when US made Landsat products available for free. In contrast, S-2 products are becoming more popular, and despite not having enough data to produce temporal analysis yet, they have a better spatial resolution to develop more precise results [22,27,28,29].

In the perspective of monitoring areas with high cloud coverage, such as tropical regions and estuarine areas, some developments have been reported in using Synthetic Aperture Radar (SAR) products [30,31,32]. To increase SAR usage products for environmental monitoring and security, the European Spatial Agency (ESA) launched the Sentinel-1 (hereafter S-1) in 2014 [33]. Similar to the S-2 products, the S-1 is available for free in the Copernicus platform and covers the entire Earth [34,35].

Recently, processes such as stacking, coregistration, and data fusion of optical with radar products have been applied to improve classification quality and its accuracy. Although radar polarization may be a barrier to identify some features, this product does not have the presence of atmospheric obstacles, such as clouds [11,27,36].

The synergetic use between optical and radar data is a recognized alternative for urban areas studies [37,38,39]. The literature indicates that although the limitations of the microdots in detecting the variety of spectral signatures over the urban environment, such data aggregation contributes to improve the classification accuracy. Also, the importance of radar data has been emphasized in tropical environmental studies [31,40,41,42], once the cloud coverage in these areas is high throughout the year hindering the use of optical images [43,44], and therefore, making radar data an alternative to acquiring imagery during all months of the year in these locations.

Another alternative described in the literature for accuracy enhancement of final classification results is the inclusion of optical indexes of vegetation, soil, water, among others [45,46,47,48]. Indexes included in the data sets allow us to increase the training data range and class statistical possibilities of classification algorithms, thereby raising their efficacy.

Besides choosing suitable products and deciding how to combine different imagery types, the developer of LULC classifications must test different methodologies to select the most accurate for each landscape analyzed [27,36,49]. In this sense, the Machine Learning (ML) algorithms are presented as optimal solutions to supervised classification, where there is no need of ground information measurements of the entire landscape to determine the different classes of LULC for the whole area. Some ML algorithms have been used to classify images with low, medium, or high spatial resolutions [50,51], among them are the Random Forest (RF) [52], Support Vector Machine (SVM) [53], Artificial Neural Networks (ANN) [54], and the k-nearest neighbor (k-NN) [55]. In addition, for the proper selection of satellite imagery, application (or not) of data fusion, and choosing the fittest classification algorithm method, we should also select computer programs that are robust enough to run each of those LULC ML classifications [50].

In this perspective, this study aims to investigate the synergy use between S-1 and S-2 products to identify the suitability for LULC based on ML classification approach. In addition, derivate products such as vegetation and water indexes and SAR textural analysis were applied in the RF classification to finally test all data sets and select the most thematic accurate product for tropical urban environments at regional spatial scale.

## 2. Satellite Data and Methods

### 2.1. Study Area

**Figure 1:**
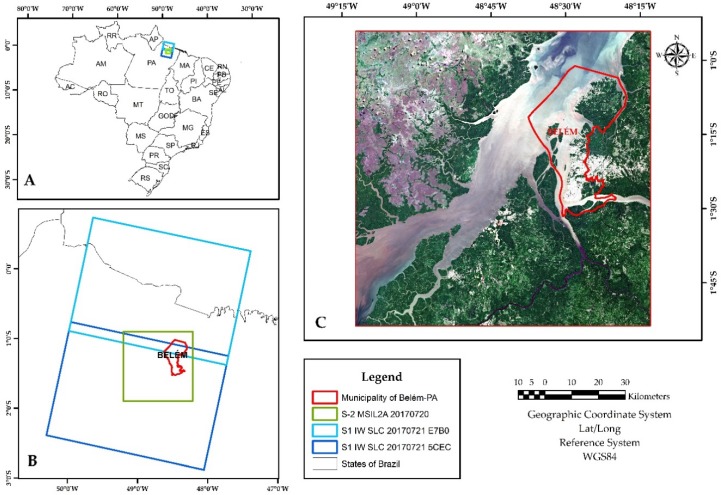
Study area in the municipality of Belém, state of Pará, Brazil *. * Where (**A**) is the location in the Brazilian territory of the S-1 and S-2 scenes used; (**B**) shows the relative tracks of the S-1 scenes and S-2 tile used; and (**C**) is an RGB composition of the S-2 scene where it is illustrated the complexity of the tropical coastal environment chosen (water bodies, different types of vegetation (dense, lowlands, and mangrove), impervious areas, among other ecosystems).

The selected study area is the city of Belém in the Eastern Amazon, Brazil. The city has a total territorial area of 1059.46 km², subdivided into eight administrative districts, all included in this study [56]. The estimated population in 2017 was 1,452,275 residents. According to the Köppen classification, the climate is tropical Afi, with average annual rainfall reaching 2834 mm [57]. The forest fragments of the locality are classified as Terra Firme and Várzea forests, being subtypes of dense Ombrophilous forests [58]. The humid tropical environments in coastal Amazon is described as a complex environment which involves the relationship of flowing rivers with the ocean, different types of natural and anthropized vegetations, and impervious surfaces [59]. Figure 1 shows the study area considered and the coverage of the selected satellite scenes.

### 2.2. Data Source and Collection

The products acquisition of both S-1 and S-2 were performed in the Copernicus open access hub platform considering the cloudy coverage of less than 5% for the S-2 product and the date proximity of the S-1 product in relation to the S-2 (one day of difference). The Planet Labs scenes were acquired through a contract of the Environment and Sustainability Secretariat of the state of Pará, Brazil.

#### 2.2.1. Sentinel-1 Images

In order to cover the whole study area, two S-1 images were collected with an S-1 C-band SAR Interferometric Wide Swath (IW) in dual polarization mode (VV + VH) from 21 July 2017. Data characteristics and the main characteristics of S-1 are described in Table 1.

#### 2.2.2. Sentinel-2 Images

One scene of S-2A Level-1C (hereafter, L1C), with radiometric and geometric corrections, was acquired for this study. The S-2A L1C provides the top of atmosphere (TOP) reflectance. The S-2A L1C has a radiometric resolution of 12 bits, a swath width of 290 km, and the wavelength of its bands range from 443 nm to 2190 nm. The spatial resolution of the bands is distributed as (i) four of 10 m, (ii) six of 20 m, and (iii) three of 60 m. The image selected has 0% of cloud cover and is from 20 July 2017. Disregarding the SWIR/Cirrus band, which was used for the atmospheric correction, all bands were used for the classification step.

#### 2.2.3. Planet Imagery

Seventeen high-resolution Planet scenes acquired in 28 July 2017 were used to validate the RF classification. Since early 2017, the sun-synchronous orbit of this satellite has the temporal resolution of one day, making it an excellent instrument for monitoring and data validation [60,61]. The specifications of the Planet mission for the images acquired are described in Table 2.

### 2.3. Data Analysis

**Figure 2:**
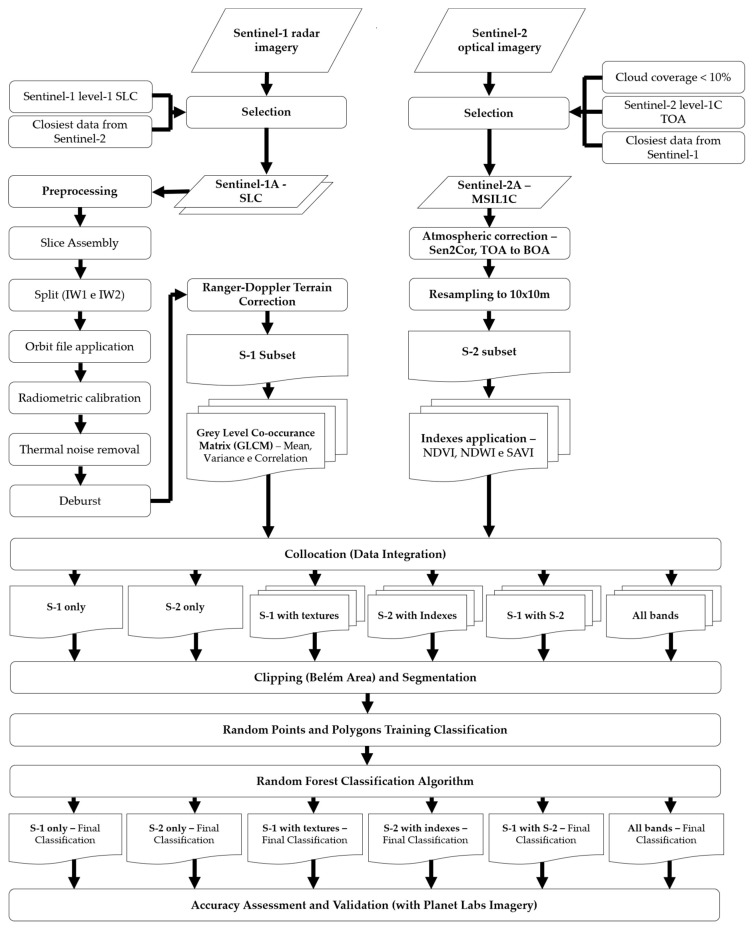
Process flowchart for Random Forest (RF) classification of Land Use and Land Cover (LULC) classes using S-1 and S-2 integration and validation with Planet Labs imagery.

The data processing is presented in the flowchart illustrated in Figure 2 and involves (i) preprocessing and data integration, (ii) product segmentation and RF classification, and (iii) accuracy assessment and validation.

#### 2.3.1. Preprocessing Data

The preprocessing of the S-2 consisted in the atmospheric correction of the data, made by Sen2Cor algorithm [36,62,63] to obtain surface reflectance. All S-2 spectral bands were resampled to 10-m spectral resolution using the bilinear upsampling method and a mean downsampling method.

As already indicated, two S-1 images were also used, and a slice assemble technique was required to join them. A split of the subswots IW1 and IW2 was applied to reduce the scene size, hence improving the processing time. The application of the orbit file, radiometric correction, thermal noise removal, and deburst was applied as it is a well-consolidated methodology. We opted not to apply a Speckle filter, using Multilooking with a single look (5 m Range looks and 20 m Azimuth looks). Finally, a range-Doppler terrain correction was applied, using the UTM WGS84 projection and the 30 m SRTM, where 10-m resampling was made to fit the integration requirements [27,36,37,64].

#### 2.3.2. Radar Textures and Multispectral Indexes

All of the derived information, for both S-1 and S-2 data, was calculated in the SNAP 6.0 software. For the S-1 product, we derived three Grey-Level Co-occurrence Matrix (GLCM), with a 5 × 5 mobile window size, in all directions based on the variogram method [65]. In general, GLCM estimates the probability of pixel values (within moving windows) co-occurring in a given direction and a certain distance in the image [66]. We computed the mean, variance, and correlation (Table 3). These GLCM statistics were applied for both VV and VH, generating a total of six products to be added in the RF classification [40,46,67]. Moreover, for the S-2 product, we estimated three different normalized radiometric indexes (NDVI-Normalized Difference Vegetation Index, NDWI-Normalized Difference Water Index, and SAVI-Soil-Adjusted Vegetation Index) (Table 3).

#### 2.3.3. Image Stacking and Image Segmentation

For the image stacking of the S-1 and S-2 products it was used the nearest neighbor resampling method. In the tool selected, two products were used, where the pixel values of one product (the slave) were resampled into the geographical raster of the other (the master) [71]. We used the S-1 product as the master and S-2 data as the slave [27,32,37,63,72]. Subsequently, and similarly, the integration was made with S-2 and vegetation and water indexes, S-1 and GLCM textural measures, and the combination of all products generated [46].

To better aggregate pixels with similar values, a segmentation procedure was performed [32,73,74]. This procedure was made using only the collocation of S-1 and S-2 products. For this, a local mutual best fitting region merging criteria was performed and a Baatz & Schape merging cost criteria selected [75,76]. A total of 82,246 segments were produced.

#### 2.3.4. Random Points’ Classification and RF Image Classification

After the segmentation process, all the other steps were developed in GIS software. Basically, 1600 random points were defined to be overlapped by the segmentation polygons, and these were visually interpreted as one of the twelve selected classes. The twelve classes were defined considering the potential attributed by the literature to RF classification algorithm and the S-1 and S-2 synergy, and the particularities of the study area [27,37]. In Table 4 it is possible to identify how the classes were interpreted with colored compositions for S-1 and S-2.

The RF is described in the literature as an ML classification algorithm where the users must choose a minimum of two parameters: (i) the number of trees to grow in the forest (N) and (ii) the depth of those trees (n) [50,52]. We implement this procedure in ArcGIS 10.4 software. The RF classifier in the ArcGIS software is named “Train Random Trees Classifier”. In this classifier, the user must input the following parameters; (i) the satellite image to be classified, (ii) the train sample file (in shapefile format), (iii) the max number of trees (N), (iv) the max tree depth (n), and (v) the max number of samples per class (parameter in which we used the default value of 1000). We also added the segment attributes of Color, Mean, Std, Count, Compactness, and Rectangularity, as they were options in the ArcGIS 10.4 RF algorithm.

To select the number of variables that provide sufficiently low correlation with adequate predictive power, tests were conducted aimed at assessing the best accuracy. All experiments were chosen proportionally with the default values proposed by Forkuor et al. [22]. The best possible scenario, in which the classification was able to run in the ArcGIS 10.4, was with 700 as maximum number of trees (N), 420 as the depth of each tree can grow (n), and 1000 as max number of samples per class; values were applied for all the six classifications made. These numbers were the most significant possible, once the literature suggests that there is no standard value for the number of trees (N) and the number of variables randomly sampled as candidates at each split (n) [50,52].

#### 2.3.5. Accuracy Assessment

As for validation assessments, we carried out a few tests to fully comprehend how the classification was defined and how good was the results found. Firstly, we investigated the mean and standard deviation of the spectral signatures for both S-1 polarizations and for all S-2 bands used for RF classification. With this assessment, we could identify which were the bands that had a greater distance (separation), in the analyzed samples. To evaluate the performance of the attributes and accuracy of the maps produced, we performed statistical approaches based on the separability means of Jeffries–Matusita (JM) and Transformed Divergence (TD), for the two polarizations of S-1 and all bands of the S-2. These coefficients are based on the Bhattacharyya distance statistics and range from 0 to 2, where values greater than 1.8 are quite distinct and values below 1.0 should be disregarded or grouped into a single class [77]. After understanding how the bands statistically separated and how different were their spectral responses, it is also possible to know and how much (algorithm decision making) they contributed to the classification. In this sense, we investigated the bands’ contribution for each LULC classification produced.

As a product of the random trees’ classifier, in ArcGIS 10.4, we analyzed the Producer’s and User’s accuracy (PA and UA, respectively). The PA and UA are prior accuracies (made by 60% of the training samples), which are produced by the trees selected in the model. We analyzed them in the six LULC classification performed.

Finally, by cross-validation performed through the collection and visual interpretation of classes (in Planet’s high spatial resolution images) of 1232 random points, we computed the overall accuracy (OA) and the Kappa coefficient. These are statistics that allow us to analyze and affirm the validity and accuracy of the results. We also ranked these OA and Kappa coefficient values from highest to lowest.

## 3. Results

**Figure 3:**
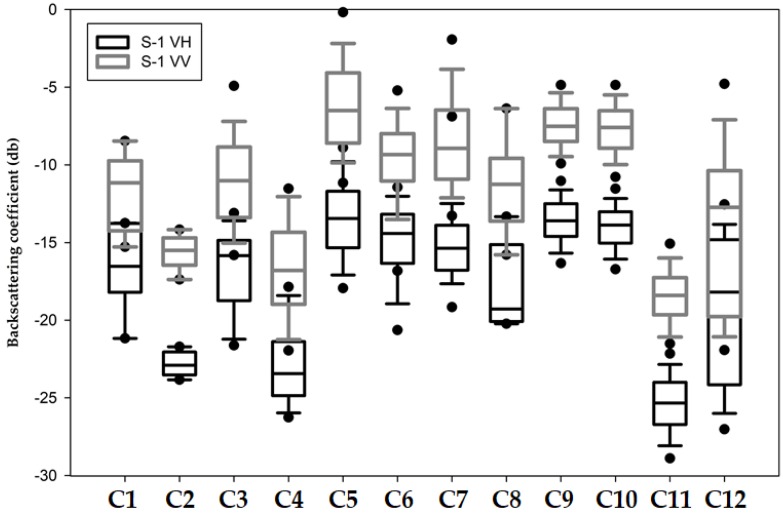
Backscattering coefficient by LULC class analyzed of VV and VH polarizations of the processed S-1 product *. * The names of the classes respect the class code of the interpretation key as follows. C1 = Agriculture; C2 = Airport; C3 = Bare Soil; C4 = Beach area; C5 = Built-up; C6 = Grassland; C7 = Highway; C8 = Mining; C9 = Primary vegetation; C10 = Urban vegetation; C11 = Water with sediments; C12 = Water without sediments.

The analysis of potentially separable classes through the VV and VH polarizations are presented in Figure 3. The separability found with low values occurs due of a superficial backscatter, highlighting in this case separation in the order of 7 dB, 6 dB, and 9 dB, for airport, beaches, and water with sediment, respectively. On the other hand, we could also identify the occurrence of backscatter double bouncing (built-up areas) and volumetric (primary and secondary vegetation). Because of the identified backscattering, the possibility of class identification through RF increases. However, for the other classes, in which the box plots significantly overlap, the likelihood of variable distinction decreases.

**Figure 4:**
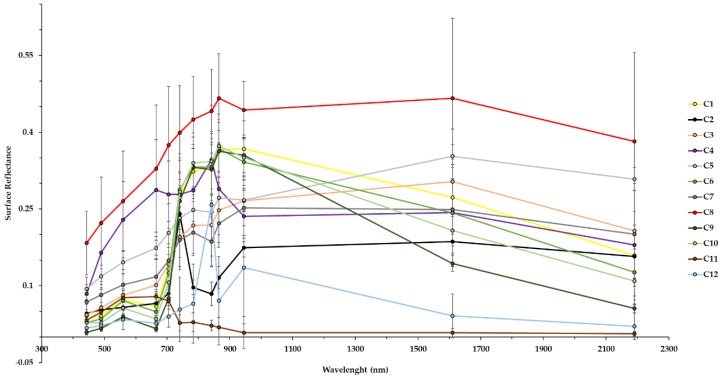
Wavelengths analysis by LULC class of S-2 bands.

For the bands explored in the analysis of separation by the spectral response (surface reflectance) of each class (Figure 4), we could see that some classes tend to get overlaid, once their spectral responses are quite similar in different wavelengths. This was the case for agriculture, grassland, primary vegetation, and urban vegetation, in which the lines generated in the dispersion have close values at different wavelengths. A high separability of classes was noted in the following classes: mining, airport, water with sediments and water without sediments. The classes that present different spectral behaviors from the others tend to perform better classification results since when applying the RF algorithm, it can produce trees and choose the variables for classification with higher precision. The analysis of these backscattering and spectral patterns allows us to provide information for further monitoring of these classes in the study area.

The JM and TD variability results are illustrated in matrix presented in Table 5. For the separability of classes in S-1, we were able to identify only good values (above 1.8) for some airport separability with other classes, for primary vegetation separability with water with sediments and water with sediments with water without sediments. On the other hand, the class separability in for the S-2 bands was significant for different classes, reaching the maximum value for both water categories and primary vegetation. These separability results reassure us that the potential of S-2 to identify a more significant number of classes is significantly better than for the S-1 to do so.

**Figure 5:**
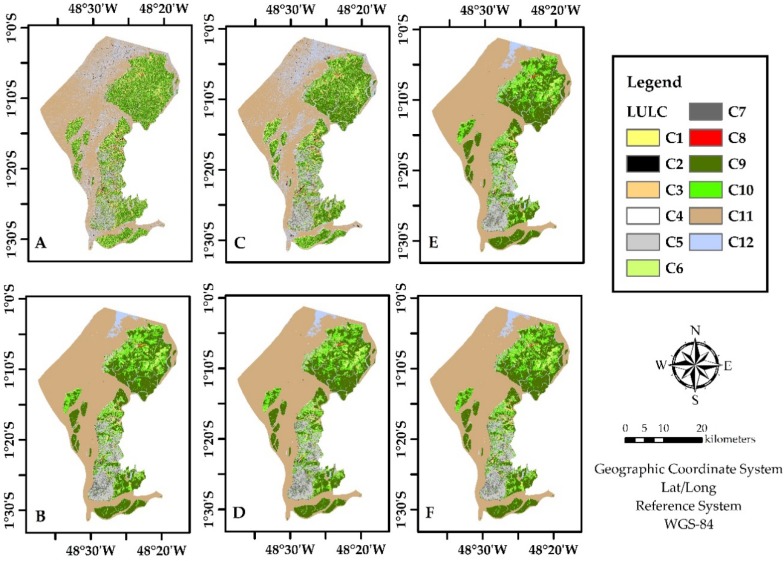
LULC classification maps produced by RFs *^#^. * Where (**A**) is for S-1 only, (**B**) is S-2 only, (**C**) is S-1 with textures, (**D**) is S-2 with indexes, (**E**) is S-1 with S-2, and (**F**) is for all bands. ^#^ The classes are represented as follows. C1 = Agriculture; C2 = Airport; C3 = Bare Soil; C4 = Beach area; C5 = Built-up; C6 = Grassland; C7 = Highway; C8 = Mining; C9 = Primary vegetation; C10 = Urban vegetation; C11 = Water with sediments; C12 = Water without sediments.

In Figure 5 it is illustrated the six LULC classification produced.

**Figure 6:**
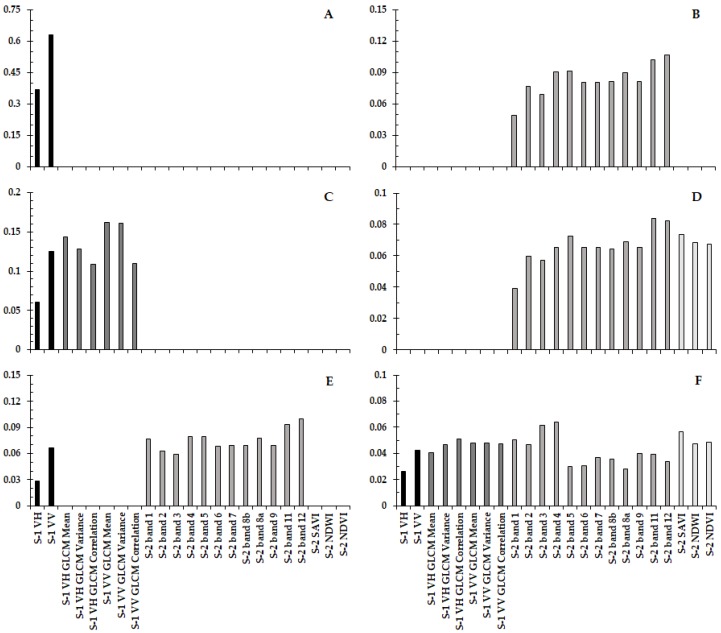
Bands contribution for RF classification *. * Where (**A**) is the band’s contribution for the S-1 only, (**B**) is for S-2 only, (**C**) is S-1 with textures, (**D**) is for S-2 with indexes, (**E**) is S-1 with S-2, and (**F**) is all bands.

The contribution of each band for the six RF classifications produced is described in Figure 6A–F. Band 12 of the S-2 product (SWIR band) was the only band that repeats, as the most significant contributor for the RF classification made (see Figure 6B,E), this band is the main contributor for the classifications of S-2 only and the integration of S-1 with S-2. Thus, in all the classifications that contain the S-2 band, some of its bands were identified as one of the most significant contributors to the classification. Therefore, for the classification with all products, the red band of S-2 had the highest contribution (0.0641), for S-1, together with S-2, and for the S-2 only, it was SWIR band 12 of S-2 (0.1 and 0.1067, respectively), and for S-2 and indexes it was the SWIR band 11 (0.084). On the other hand, for the classifications that only consider the SAR products, it was shown that the largest contribution was from S-1 VV (0.6307), and for S-1 and its textures, it was the S-1 VH GLCM Mean (0.1434).

The PA and UA results are presented in Table 6. The optical integration with radar and the optical only classifiers stands out with generally better results of PA. The worst results were found for classifiers without the S-2, being S-1 only and S-1 with GLCM secondary products. The UA followed a similar trend. The worst result by class was found for the agriculture and mining classes in the classifier that used radar and its textures, where the results for both PA and UA were equal to 0%. However, the mining class achieved 100% in PA for all classifiers that had S-2 bands. S-2 only, S-2 with its indexes, and the integration of S-1 with S-2 had more than one PA equal to 100%; Agriculture (C1) and mining (C8) were repeated in all these classifications, for the integration of S-1 and S-2, the Airport (C2) class also had PA equals to 100%. The only UA with 100% was for the identification of beaches in the classification S-2 with indexes.

Table 7 illustrates the OA and the Kappa coefficients found in this research. It is possible to understand that the integration of the S-1 and S-2 products resulted in a more precise product (91.07% of OA and 0.8709 of Kappa) and, as expected, the S-1 product alone had the worst result of all the analyses (56.01% of OA and 0.4194 of Kappa). The inclusion of textures in the S-1 products increased the results of the RF classification (61.61% OA and 0.4870 of Kappa), on the other hand, the inclusion of vegetation and water indexes in the S-2 product reduced its OA (89.45%) and Kappa coefficient (0.8476) when compared with the S-2 alone (89.53% of OA and 0.8487 for the Kappa coefficient). The integration of all the products analyzed produced the worst result among all the data combination that have S-2 products involved (87.09% OA and 0.8132 of Kappa coefficient). However, the results of OA and Kappa coefficient for the four best classifications were similar.

## 4. Discussion

Among the ML methods described in the literature [50], SVM and RF stand out for their good classification accuracy. These methods usually have good results when compared with similar methods such as k-NN, or more sophisticated ones, such as ANN and Object Based Image Analysis (OBIA) methods [27,36,49]. Since these two types of ML algorithms are the most outstanding, we analyzed the discussion considering some papers that used both algorithms.

We verified that the application of optical radiometric indexes and radar textures is widely accepted as mean to improve ML classification [11,27,36,46,63,78]. However, our OA result, when considering all bands in the classification, was lower than for S-1 and S-2, S-2 only, and S-2 with indexes. These results show that the insertion of the data derived from the optical image did not have a significant impact on the final classification, whereas the SAR product data, which improved the classification when considering S-1 only, contributed to the classification, but not enough to raise OA when all bands were considered. Some authors argue that major classification enhancements occur only when we insert primary data into the dataset, such as SRTM [63,64,79].

The lowest OA values found were for S-1 products; this is in agreement with what is found in the literature [27,36,40,46,80]. SAR products, while having the advantage of penetrating the clouds and always giving views consistent with the Earth’s surface, fail when forced to distinct a vast amount of features (classes) [80,81]. In our study, this is noticeable in Table 5, in which JM and TD values were lower than 1.8 in almost all categories, and this can be seen in local studies on the Brazilian Amazon coast, even using radar images with better quality than those of S-1 [82,83]. Discrimination of a large number of classes on the radar is possible only through the application of advanced techniques, such as SAR polarimetry [84].

Maschler et al. [85], in an application of the RF classifier with high spatial resolution data of 0.4 m (Hyspex VNIR 1600 airborne data), obtained excellent separability of the classes in the electromagnetic spectrum. From this good separability, they were also able to produce an excellent OA of 91.7%. This separability and these good results can be identified in our study too. The possibility of separating the classes from the insertion of the training and validation samples is fundamental for the satisfactory production of the results for LULC classification.

Among the authors who applied several ML methods (RF, SVM, and k-NN), Clerici et al. [36] consider the data fusion of S-1 and S-2 products. They also applied radiometric vegetation indexes (NDVI, Sentinel-2 Red-Edge Position index (S2REP), Green Normalized Difference Vegetation Index (GNDVI), and Modified SAVI) and textural analysis of the S-1, in order to interpret their contributions to the supervised classification accuracy. Six classes were tested after segmentation to have similar pixel values. Their results were considerably worse than ours. In their image stacking, they found an OA of 55.50% and a Kappa coefficient of 0.49. The isolated results of S-1 and S-2 were also worse than our integration. They suggest SVM for their study area because it presents better accuracy results.

Whyte et al. [27] applied RF and SVM algorithms for LULC classification in the synergy of S-1 and S-2. These authors used the eCognition image processing software to produce segments and the ArcGIS 10.3 for RF and SVM classifications. They applied derivatives from both S-1 and S-2 to test the data combination for LULC. They selected 15 classes and found better results when using all products (S-1, S-2, and their derivatives) using the RF algorithm. The OA was 83.3% and the Kappa coefficient was 0.72. However, all the scenarios of synergy appeared to have higher results than using optical data only. This study contrast to what we found, once our results with derivatives were worse in almost all circumstances, except in S-1 and GLCM stacking.

Zhang & Xu [49] also applied the fusion test of optical images and radar for multiple classifiers (RF, SVM, ANN, and Maximum Likelihood). Optical images of Landsat TM and SPOT 5 were used, while SAR images were of ENVISAT ASAR/TSX. The authors could interpret that the best values were found for the RF and SVM classifications, while the fusion of optical and SAR data contributed to the improvement of the classification, increasing the accuracy by 10%.

Deus [46] used the synergy of ALOS/PALSAR L band and Landsat 5 TM and applied several vegetation indexes and SAR textural analysis. The author applied the SVM algorithm in order to obtain five LULC classes. Their highest OA was 95% when only the features with the best performance in the classification were combined, including both PALSAR and TM bands and their derivatives.

Jhonnerie et al. [86] and Pavanelli et al. [40] also used ALOS/PALSAR and the Landsat family. They considered LULC classes 8 and 17, respectively. Both studies applied the RF algorithm. While the first authors used the ERDAS image application for RF classification, the second authors used the R software for their image classification. They applied vegetation indexes and GLCM textural analysis. For the RF classification, both authors found their highest OA and Kappa coefficient results for the hybrid model, 81.1% and 0.760 [86] and 82.96% and 0.81 [40].

Erinjery et al. [11] also used the synergy between S-1 and S-2, and their derivatives to compare results from two ML, including Maximum Likelihood and RF. The total number of classes was seven. For the RF classification, an OA of 83.5% and a Kappa coefficient of 0.79 were found. They state the inclusion of SAR data and textural features for RF classification as a tool to improve the classification accuracy. Similarly to our study, they found an OA lower than 50% when using only the S-1 product.

Shao et al. [78] integrated the S-1 SAR imagery with the GaoFan optical data to apply a RF classification algorithm in six different LULC classes. They also produce SAR GLCM textures and vegetation indexes. Their best result was obtained considering all the features stacked. An OA of 95.33% and a Kappa coefficient of 0.91 was obtained. The use of S-1 only had the worst result, with an OA of 68.80% and a Kappa coefficient of 0.35.

Haas & Ban [37], in a data fusion analysis of S-1 and S-2, applied the SVM classification method. Considering 14 classes, they found an OA of 79.81% and a Kappa coefficient of 0.78. The authors suggested the postclassification analysis to improve its accuracy once their final objective was to have area values to apply the benefits transfer method of Burkhard et al. [17] to environmentally evaluate the urban ecosystem services of an area. The number of classes was similar to our study, and it was possible to check that the accuracy was superior.

In regional studies [2], and for global applications [87,88] of RF classification, the results found of OA were below those of ours. Their OA results were 75.17% [88], 63% for South America using RF [87], and 76.64% for Amazon LULC classification [2]. All these studies used satellite data with a temporal resolution of 16 days and a spatial resolution of 30 m. In contrast, we found results with a revisit time of five days for optical and six days for SAR, and with a greater spatial resolution (10 m). Also, we produced a dataset with more classes (12) against six [87] and seven [88] classes found for global studies.

## 5. Conclusions

In this work, the best result found was in the integration of S-1 and S-2 products. In general, integrating the vegetation and water indexes and SAR textural features made the OA and kappa coefficient decrease. The worse result was found for the S-1 only classification. The results encountered agreed in its most part with the literature. For our better classifications, the OA results were significantly greater than what is found in the literature for global and Amazon applications of RF classification.

In both the PA and UA scenarios and the Kappa statistic scenario it can be stated that the integration of S-1 and S-2 presented better results in the implemented ML technique. However, if on the one hand the GLCM increased the SAR product accuracy, on the other hand, the inclusion of vegetation and water indexes decreased the optical accuracy, when compared with the single use of S-1 and S-2, respectively. Lastly, the results found for the integration of all products were worse than the ones observed for the combination of S-1 and S-2 only.

Depending on the final aim of the LULC classification, it could be relevant to make a postclassification analysis because many spectral responses resemble each other and can confuse the ML process. However, in the best scenario produced, the accuracy found was satisfactory for several types of analysis. Furthermore, it is possible to use the data integration of S-1 and S-2 to LULC cover classification in tropical regions. It is noteworthy that few studies with similar methodology were found in the literature for the southern hemisphere.

In this sense, the research findings can contribute to current knowledge on urban land classification since methodology applied produces more accurate local data. Furthermore, the present results were obtained by using a smaller revisit period and a higher number of land classes than previous global studies which considered our study area. Future work must be done with S-1 and its variables; once in tropical regions it is difficult to have long terms of optical data available. Finally, we encourage the synergetic use of S-1 and S-2 for LULC classification, considering the availability on near date.

## Figures and Tables

**Table 1:** Main attributes from the Synthetic Aperture Radar (SAR) dataset.

**S-1**	**Operation**	Since 03/04/2014-current	**SAR data**	**Imaging date**	21/07/2017
	**Orbit height**	693 km		**Swath width**	250 km
	**Inclination**	98.18°		**Sub-swaths**	3
	**Wavelength**	C-band (3.75–7.5 cm)		**Incidence angle range**	29.1–46.0°
	**Polarization**	Dual (VV + VH)		**Spatial resolution**	5 × 20 m (single look)
	**Temporal resolution**	Six days		**Pixel Spacing**	2.3 × 17.4 m

**Table 2:** Main attributes of Planet Labs imagery and the scene selected.

**Temporal resolution**	Daily	
**Swath width**	24.6 km × 16.4 km	
**Orbit Altitude (km)**	475 (~98° inclination)	
**Cloud cover of the scenes**	0%	
**Radiometric resolution**	12 bits	
**Operation**	Daily imagery since 14/02/2017-current	
**Imaging date**	28/07/2017	
**Spectral Bands**	**Wavelength (nm)**	**Spatial Resolution (m)**
Blue	455–515	3
Green	500–590	3
Red	590–670	3
NIR	780–860	3

**Table 3:** Textural analysis and radiometric indexes employed in this research.

S-2 Indexes		References
Index Applied	Equation *	
NDVI	$\frac{\mathrm{NIR}-\mathrm{Red}}{\mathrm{NIR}+\mathrm{Red}}$	[68]
NDWI	$\frac{\mathrm{NIR}-\mathrm{MIR}}{\mathrm{NIR}+\mathrm{MIR}}$	[69]
SAVI	$\frac{L\times (\mathrm{NIR}-\mathrm{Red})}{\mathrm{NIR}+\mathrm{Red}+0.5}$	[70]
**S-1 GLCM Textural Measures**		
Mean	$\overset{N-1}{\underset{i,j=0}{\sum }}{\mathrm{iP}}_{i,j}$	[46]
Variance	$\overset{N-1}{\underset{i,j=0}{\sum }}{\mathrm{iP}}_{i,j}\left(i-\mu \right)²$	
Correlation	$\frac{\sum_{i,j=0}^{N-1}{\mathrm{iP}}_{i,j}-\mu_{x}\mu_{y}}{\sigma_{x}\sigma_{y}}$	

* Where NIR is the near infrared band, 842 nm for S-2, for NDVI, NDWI, and SAVI; Red is 665 nm for S-2 for NDVI and SAVI; MIR (Medium Infrared) is 2190 nm for S-2, for NDWI; P(i,j) is a normalized gray-tone spatial dependence matrix such that SUM(i,j = 0, N − 1) (P(i,j)) = 1; i and j represent the rows and columns, respectively, for the measures of Mean, Variance and Correlation; μ is the mean, for the Variance textural measure; and N is the number of distinct grey levels in the quantized image; μ_x_. μ_y_, σ_x_, and σ_y_ are the means and standard deviations of p_x_ and p_y_, respectively, for the correlation textural measure.

**Table 4:** Keys of interpretation to recognize the different LULC with S-1 and S-2 colored compositions.

Classes	Class Code	S-1 (R: VV; G: VH; B: VV/VH) *	S-2 (R: B4; G: B3; B: B2) *	S-2 (R: B12; G: B8; B: B4) *
Agriculture	C1	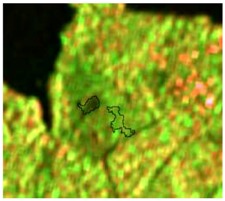	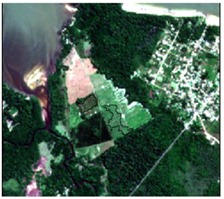	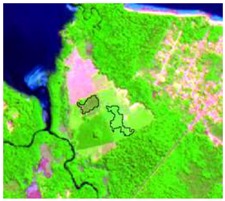
Airport	C2	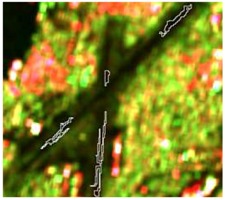	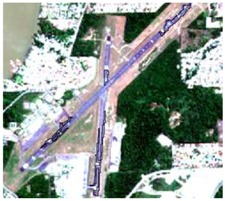	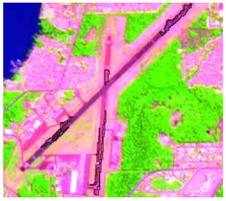
Bare Soil	C3	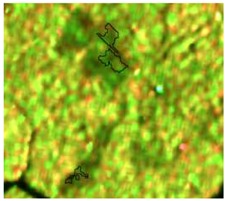	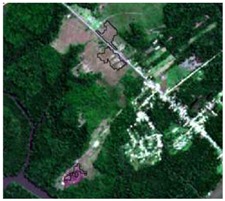	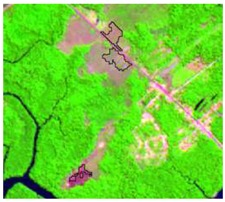
Beach	C4	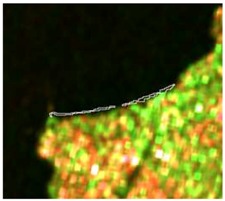	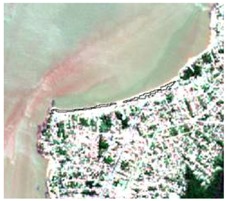	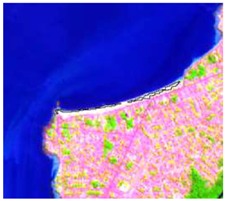
Built-up	C5	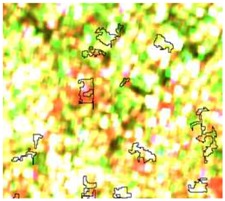	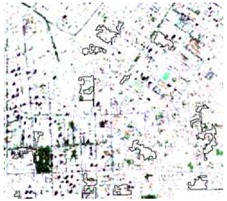	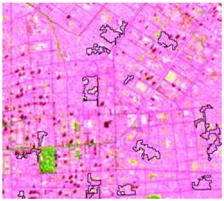
Grassland	C6	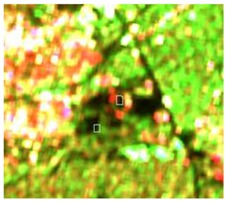	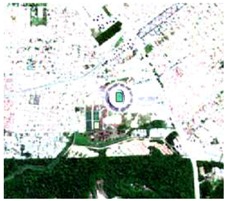	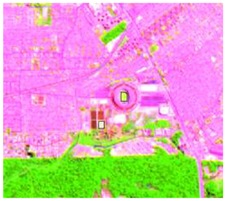
Highway	C7	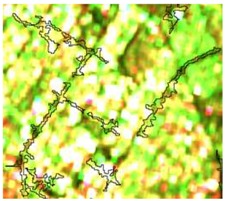	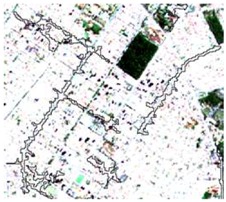	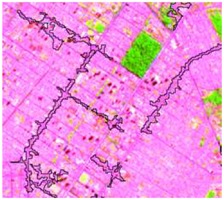
Mining	C8	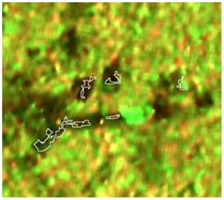	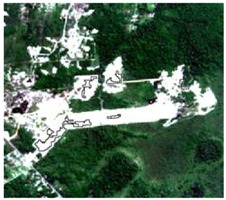	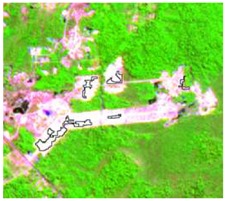
Primary Vegetation	C9	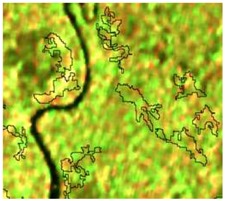	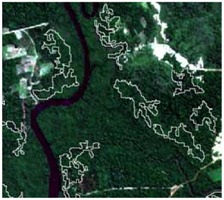	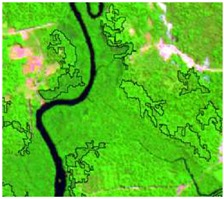
Urban Vegetation	C10	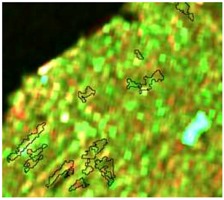	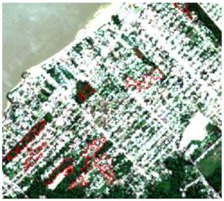	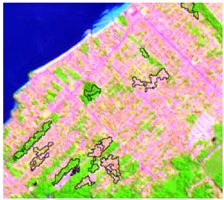
Water with Sediments	C11	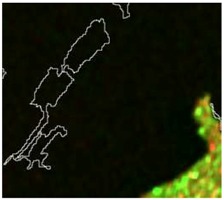	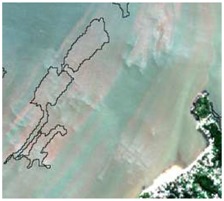	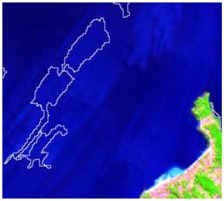
Water without Sediments	C12	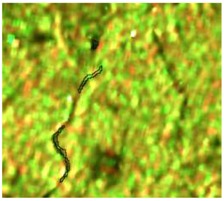	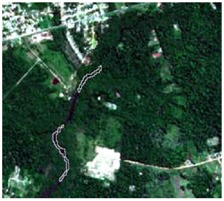	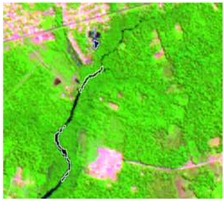

* The drawn polygons were the ones produced in the segmentation and classified for each class to perform the LULC classification.

**Table 5:** JM and TD * variability results for the similarity of each class. Values in blue represents the variability for S-1 and values in green is for the S-2 *^#^.

	C1	C2	C3	C4	C5	C6	C7	C8	C9	C10	C11	C12
**C1**	-	0.00	0.00	0.00	0.00	0.00	0.00	0.00	0.00	0.00	0.00	0.00
**C2**	0.00	-	0.00	0.00	0.00	0.00	0.00	0.00	0.00	0.00	0.00	0.00
**C3**	0.00	1.85	-	0.00	1.73	1.92	1.73	0.00	2.00	1.84	2.00	2.00
**C4**	0.00	1.52	0.83	-	0.00	0.00	0.00	0.00	0.00	0.00	0.00	0.00
**C5**	0.00	1.94	0.86	1.50	-	0.00	1.53	0.00	2.00	1.95	2.00	2.00
**C6**	0.00	1.91	0.51	1.19	0.99	-	1.99	0.00	1.98	1.67	2.00	2.00
**C7**	0.00	1.91	0.54	1.27	0.10	0.73	-	0.00	2.00	1.92	2.00	2.00
**C8**	0.00	1.96	0.74	1.12	1.52	0.64	1.33	-	0.00	0.00	0.00	0.00
**C9**	0.00	2.00	0.92	1.76	1.19	0.28	0.99	1.20	-	1.78	2.00	2.00
**C10**	0.00	1.98	0.52	1.49	0.46	0.69	0.30	1.29	0.72	-	2.00	2.00
**C11**	0.00	1.33	1.30	0.43	1.70	1.45	1.55	1.31	1.83	1.79	-	0.18
**C12**	0.00	1.46	0.98	0.41	1.53	0.94	1.33	0.77	1.36	1.47	1.99	-

* Where the values range from 0 to 2, the higher the value, the better the chance of the class separating into the LULC classification. ^#^ The classes are represented as follows. C1 = Agriculture; C2 = Airport; C3 = Bare Soil; C4 = Beach area; C5 = Built-up; C6 = Grassland; C7 = Highway; C8 = Mining; C9 = Primary vegetation; C10 = Urban vegetation; C11 = Water with sediments; C12 = Water without sediments.

**Table 6:** PA and UA for each class in the different types of RF classifications produced *.

Class Code	S-1 Only		S-2 Only		S-1 with Textures		S-2 with Indexes		S-1 with S-2		All Bands	
	PA (%)	UA (%)	PA (%)	UA (%)	PA (%)	UA (%)	PA (%)	UA (%)	PA (%)	UA (%)	PA (%)	UA (%)
**C1**	50.0	7.7	100.0	18.2	0.0	0.0	100.0	20.0	100.0	20.0	50.0	20.0
**C2**	60.0	16.7	80.0	66.7	40.0	25.0	80.0	80.0	100.0	50.0	80.0	57.1
**C3**	31.2	23.8	56.3	64.3	25.0	21.1	56.3	69.2	65.6	75.0	46.9	48.4
**C4**	40.0	21.1	70.0	77.8	80.0	47.1	80.0	100.0	80.0	88.9	40.0	66.7
**C5**	41.8	42.2	75.5	85.1	53.1	51.5	79.6	86.7	80.6	94.1	71.4	88.6
**C6**	27.6	10.3	58.6	60.7	41.4	16.2	51.7	55.6	58.6	63.0	55.2	48.5
**C7**	12.1	9.3	75.8	59.5	15.2	11.9	78.8	59.1	78.8	70.3	69.7	46.0
**C8**	20.0	4.0	100.0	55.6	0.0	0.0	100.0	50.0	100.0	50.0	100.0	62.5
**C9**	46.9	65.7	89.2	94.6	60.7	74.3	88.8	94.3	90.3	94.0	88.8	93.1
**C10**	16.7	18.1	68.6	64.2	18.6	21.6	66.7	62.4	73.5	70.8	60.8	60.2
**C11**	41.4	8.6	93.1	81.8	48.3	9.4	93.1	77.1	93.1	75.0	79.3	67.7
**C12**	75.3	98.5	99.5	99.7	77.2	98.7	99.2	99.7	99.5	99.7	99.0	98.7

* The classes are represented as follows. C1 = Agriculture; C2 = Airport; C3 = Bare Soil; C4 = Beach area; C5 = Built-up; C6 = Grassland; C7 = Highway; C8 = Mining; C9 = Primary vegetation; C10 = Urban vegetation; C11 = Water without sediments; C12 = Water with sediments.

**Table 7:** OA and Kappa coefficient for each classifier ranked in order of accuracy.

Data Combination	Overall Accuracy (%)	Kappa Coefficient	Rank
S-1 with S-2	91.07	0.8709	1
S-2 Only	89.53	0.8487	2
S-2 with Indexes	89.45	0.8476	3
All	87.09	0.8132	4
S-1 with Textures	61.61	0.4870	5
S-1 Only	56.01	0.4194	6
